# Impact of COVID-19 on digestive system: prevalence, clinical characteristics, outcome, and relation to the severity of COVID-19

**DOI:** 10.1186/s43162-022-00132-w

**Published:** 2022-05-21

**Authors:** Walaa M. Hashem, Heba Abdelaziz, Dina E. Sallam, Moamen Abdelfadil Ismail, Ahmed Elmetwally Ahmed

**Affiliations:** 1 Gastroenterology, Hepatology and Internal Medicine Department, Ain Shams University, Cairo, Egypt; 2 Public Health Department, National Hepatology and Tropical Medicine Research Institute, Cairo, Egypt; 3 Department of Pediatrics and Pediatric nephrology, Ain Shams University, Cairo, Egypt; 4 Internal Medicine Department, Helwan University, Cairo, Egypt

**Keywords:** COVID-19, Severe acute respiratory syndrome coronavirus 2, Gastrointestinal symptoms, Diarrhea, Outcome, Mortality

## Abstract

**Background:**

Coronavirus disease 2019 (COVID-19) is commonly associated with respiratory symptoms. However, gastrointestinal (GI) symptoms are increasingly recognized in COVID-19 patients. The aim is to study the prevalence and features of gastrointestinal manifestations in severe acute respiratory coronavirus 2 (SARS-CoV-2) infected patients and evaluate the outcome among the studied population.

**Results:**

We enrolled adult patients with laboratory-confirmed COVID-19 admitted to Ain Shams University designated hospitals, Cairo, Egypt, from March 2021 to June 2021. The patients were assigned to a GI group and a non-GI group based on the presence or absence of one or more digestive symptoms. A total of 300 hospitalized COVID-19 patients were included, of which 104 (34.7%) had one or more digestive symptoms. They were compared with 196 COVID-19 patients without GI symptoms. The most common reported GI symptom was diarrhea (82.7%). GI symptoms’ presence was higher in moderate cases. Patients with digestive symptoms presented for care later than those without (7.9±3.8 vs 7.4±7.2 days, *P*=0.5). Moreover, they have lower mortality, though non-significant (7.7 vs 12.8%, *P*=0.18). Patients with digestive symptoms had lower total leucocytic count (TLC), neutrophil count, neutrophil to lymphocyte ratio (NLR), platelet count, and higher serum sodium than those without digestive symptoms.

**Conclusion:**

GI symptoms are prevalent among COVID-19 patients, and the most common was diarrhea. The presence of GI manifestations was not associated with increased mortality.

## Background

The current outbreak of coronavirus disease 2019 (COVID-19) is responsible for the present global pandemic, which is caused by severe acute respiratory syndrome coronavirus 2 (SARS-CoV-2), a newly emergent coronavirus, that was first recognized in Wuhan, Hubei Province of China in early December 2019 [1]. In March 2020, COVID-19 spread to 166 other countries with a declaration as a pandemic by the World Health Organization (WHO) [2].

COVID-19 is primarily a respiratory disease generating a wide spectrum of symptoms, ranging from asymptomatic patients to critically ill patients with respiratory failure, shock, or multiorgan failure, passing through having mild to moderate symptoms of pneumonia, and to severe patients with dyspnea and hypoxia [3].

However, reports from both the source outbreak of COVID-19 in China and emerging data from other international sites have notified subgroups of COVID-19 patients with gastrointestinal (GI) symptoms [4] that can occur with and without pulmonary manifestations of COVID-19 [5]. The reported prevalence of GI symptoms has been highly variable ranging from 3.8 to 61.3% [6–12].

Research showed that angiotensin-converting enzyme 2 (ACE-2) is the receptor for SARS-CoV-2. ACE-2 is not only expressed in alveolar type II cells of the lung [13] but also in intestinal epithelial cells, and its expression in the intestine is more than four times that of other tissues [14]. Given that viral nucleic acid was detected in patients’ stool samples, raising the possibilities that SARS-CoV-2 could be also transmitted by the feco-oral route causing GI infection [15].

The most frequently reported GI symptom with COVID-19 is diarrhea ranging from 3 to 30% [16]. The possible underlying mechanisms for GI involvement are the direct invasion of GI epithelial cells leading to malabsorption, unbalanced intestinal secretion, and an activated enteric nervous system causing diarrhea [17]. Other mechanisms include antibiotic-associated diarrhea, changes in the composition and function of digestive tract flora, and indirect inflammatory damage [18].

Limited data exist regarding the prevalence and potential gastrointestinal implications of COVID-19 among the Egyptian population. Therefore, we aimed to assess the prevalence and clinical features of digestive manifestations in patients hospitalized with COVID-19 and to evaluate the association between digestive symptoms and laboratory results and outcomes among the studied population.

## Methods

A cross-sectional study based on identifying COVID-19 cases admitted to Ain Shams University designated hospitals, Cairo, Egypt.

The study was conducted over 4 months duration from March 2021 through June 2021, enrolling 300 adult patients “≥18 years” with laboratory-confirmed COVID-19 based on real-time reverse transcriptase-polymerase chain reaction (PCR) assay for nasopharyngeal and oropharyngeal swab specimens. We further categorized the patients into two groups based on the presence of GI symptoms: the GI group and non-GI group.

COVID-19 severity was classified as moderate, severe, or critical according to WHO guidelines [19]. Moderate cases are patients with clinical signs of pneumonia and pneumonic manifestations that can be seen in chest imaging, but no signs of severe pneumonia including SpO_2_ ≥90% on room air. Severe cases include patients with clinical signs of pneumonia and one of the following: respiratory rate >30 breaths/minute, severe respiratory distress, or SpO_2_ <90% on room air. Critical cases are patients meeting any of the following criteria: occurrence of respiratory failure requiring mechanical ventilation, presence of septic shock, and multi-organ failure that requires monitoring and treatment in the intensive care unit (ICU).

Patients who were unable to provide a history of presenting illness, with mild COVID-19 disease severity and with organic gastrointestinal diseases (e.g., peptic ulcer disease, inflammatory bowel disease), were excluded from the study.

An informed consent was obtained from all patients before enrollment. The study was approved by the Ethical Review Board of Ain Sham University (Reference Number: FMASU R 55/2020). The study protocol conformed to the ethical guidelines of the Declaration of Helsinki.

### Data collection

The study population was subjected to a well-designed data sheet covering the following topics: demographic data as (age and gender), clinical data as (digestive, respiratory, and constitutional symptoms), comorbidities, vital signs at presentation, baseline laboratory and radiological investigations, inpatient medications, and clinical outcomes.

Baseline investigations included complete blood count (CBC), neutrophil to lymphocyte ratio (NLR), alanine aminotransferase (ALT), aspartate aminotransferase (AST), serum total and direct bilirubin, serum albumin, international normalization ratio (INR), serum creatinine, sodium (Na), potassium (K), serum ferritin, C-reactive protein (CRP), lactate dehydrogenase (LDH), erythrocyte sedimentation rate (ESR), D-dimer, and cardiac enzymes. A high-resolution computed tomography (HRCT) chest was done to determine the COVID-19 Reporting and Data System (CORADs).

Data were recorded both electronically and in the patient report form. The date of symptom onset, length of hospital stay, and the severity of the patient’s condition were also recorded.

### Outcome

We monitored the clinical outcome of COVID-19 patients in both groups whether discharge, transfer to ICU, or death. The criteria for hospital discharge were 10 days after symptom onset and resolution of fever for at least 3 days without the use of fever-reducing medications and with the improvement of other symptoms.

### Statistical analysis

Data were analyzed using IBM SPSS statistics version 24. Numerical data were expressed as mean and standard deviation. Qualitative data were expressed as frequency and percentage. Chi-square test (Fisher’s exact test) was used to examine relations between qualitative variables. For quantitative data normally distributed, a comparison between paired data was done using paired *t* test (parametric *t* test). The test is considered statistically significant when *P* < 0.05.

## Results

### Demographic and clinical characteristics

A total of 300 patients with confirmed COVID-19 admitted to Ain Shams University designated hospitals were included. The mean age of the total sample was 56.95±14.3 years, and 165 (55%) of them were males. Hypertension was the most common comorbidity (45.7%) followed by diabetes mellitus (36.3%). Chronic liver disease was reported in 16 (5.3%) patients (Table 1).

**Table 1:** Demographics of patients with COVID-19

Characteristics	All patients *N*=300		GI group *N*=104		Non- GI group *N*=196		*P* value
Age, mean ± SD	56.95 ± 14.3		54.0± 14.4		58.5± 14.1		0.009*
Gender, *n* (%)							0.4
Male	165	(55)	54	(51.9)	111	(56.6)	
Female	135	(45)	50	(48.1)	85	(43.4)	
Comorbidities, *n* (%)							
Diabetes mellitus	109	(36.3)	33	(31.7)	76	(38.8)	0.22
Hypertension	137	(45.7)	42	(40.4)	95	(48.5)	0.12
Cardiovascular disease	61	(20.3)	16	(15.4)	45	(23)	0.12
Pulmonary disorders	24	(8)	10	(9.6)	14	(7.1)	0.45
Neuropsychiatric disorders	23	(7.7)	6	(5.8)	17	(8.7)	0.37
Renal disease	20	(6.7)	7	(6.7)	13	(6.6)	0.97
Endocrinal disorders	9	(3)	3	(2.9)	6	(3.1)	0.62
Hematological disorders	3	(1)	1	(1)	2	(1)	0.72
Rheumatological & immunological disorders	16	(5.3)	6	(5.8)	10	(5.1)	0.81
DVT	4	(1.3)	1	(1)	3	(1.5)	0.57
Chronic liver disease	16	(5.3)	3	(2.9)	13	(6.6)	0.17
Malignancy	20	(6.7)	8	(7.7)	12	(6.1)	0.6
Severity, *n* (%)							0.32
Moderate	294	(98.0)	103	(99.0)	191	(97.4)	
Severe	6	(2.0)	1	(1.0)	5	(2.6)	

*Significant

Regarding COVID-19, 294 (98%) had moderate disease, and 6 (2%) had severe disease. The most common symptoms were fever (80%), cough (68.7%), and dyspnea (65%). Based on the presence of GI symptoms, the patients were classified into a GI group (*n*=104) and non-GI group (*n*=196).

Among the 104 patients with GI symptoms, 11 (10.58%) patients had digestive symptoms only, and 93 (89.4%) were presented with both digestive and respiratory symptoms. Out of 196 patients, 176 (89.8%) developed respiratory symptoms alone and 20 (10.2%) patients presented with constitutional symptoms alone. There were two patients in the non-GI group who had been reinfected with COVID-19. There were similar comorbidities between the two groups (Tables 1 and 2).

**Table 2:** Treatment of patients with COVID-19

Characteristics	All patients *N*=300	GI group *N*=104		Non-GI group *N*=196		*P* value	
Treatment, *n* (%)							
Colchicine	26	(8.7)	10	(9.6)	16	(8.2)	0.67
Antiviral	78	(26.0)	32	(30.8)	46	(23.5)	0.2
HQ	69	(23.0)	31	(29.8)	38	(19.4)	0.04*
Remdesivir	13	(4.3)	3	(2.9)	10	(5.1)	0.41
Anti-IL6	10	(3.3)	5	(4.8)	5	(2.6)	0.32
Macrolides	180	(60.0)	59	(56.7)	121	(61.7)	0.38
Cephalosporines	155	(51.7)	48	(46.2)	107	(54.6)	0.21
Penicillin	2	(0.7)	2	(1.9)	0	(0.0)	0.05
Quinolones	42	(14.0)	18	(17.3)	24	(12.2)	0.22
Carbapenems	155	(51.7)	50	(48.1)	105	(53.6)	0.37
Linezolid	138	(46.0)	47	(45.2)	91	(46.4)	0.83
Acyclovir	2	(0.7)	1	(1.0)	1	(0.5)	0.65
Antifungal	10	(3.3)	4	(3.8)	6	(3.1)	0.72
Racecadotril	1	(0.3)	1	(1.0)	0	(0.0)	0.17
Rifaximin	11	(3.7)	10	(9.6)	1	(0.5)	0.000**
Metronidazole	21	(7.0)	21	(20.2)	0	(0.0)	0.000**
Nanazoxid	22	(7.3)	22	(21.2)	0	(0.0)	0.000**

*Significant

**Highly significant

The respiratory rate on admission ranged from 14 to 42 breaths/minute. A total of 206 patients presented with cough, most of them (56.3%) had dry cough with an average duration of 10.96±5.56 days.

### Clinical characteristics of patients with gastrointestinal symptoms

Gastrointestinal symptoms were observed in 104 (34.7%) patients. The most frequently reported GI manifestations were diarrhea (82.7%), vomiting (17.3%), nausea (15.4%), and abdominal pain (14.4%). Patients with GI symptoms reported significantly higher frequency of fever (88.5% vs 75.5%, *P* = 0.008), anosmia (12.5% vs 3.1%, *P* = 0.001), and bony-aches and myalgia (53.8% vs 38.8%, *P* = 0.01). Ninety-nine percent of patients with GI symptoms had moderate COVID-19, with fewer mortality (7.7%); (Tables 1, 3, and 4).

**Table 3:** Clinical characteristics of patients with COVID-19

Characteristics	All patients *N*=300		GI group *N*=104		Non-GI group *N*=196		*P* value
Symptoms, *n* (%)							
Fever	240	(80.0)	92	(88.5)	148	(75.5)	0.008*
Anosmia	19	(6.3)	13	(12.5)	6	(3.1)	0.001*
Cough	206	(68.7)	76	(73.1)	130	(66.3)	0.23
Dyspnea	195	(65.0)	71	(68.3)	124	(63.3)	0.39
Chest pain	5	(1.7)	1	(1.0)	4	(2.0)	0.49
Runny nose	5	(1.7)	3	(.9)	2	(1.0)	0.23
Sore throat	32	(10.7)	11	(10.6)	21	(10.7)	0.97
Bony aches, myalgia	132	(44.0)	56	(53.8)	76	(38.8)	0.01*
Low back pain	2	(0.7)	2	(0.7)	0	(0.0)	0.05
Headache	19	(6.3)	10	(9.6)	9	(4.6)	0.09
Lethargy, fatigue & malaise	21	(7.0)	7	(6.7)	14	(7.1)	0.89
Dryness of mouth	2	(0.7)	2	(1.9)	0	(0.0)	0.05
Metallic taste	1	(0.3)	1	(1.0)	0	(0.0)	0.17
Hoarseness of voice	1	(0.3)	0	(0.0)	1	(0.3)	0.47
Gastrointestinal symptoms, *n* (%)							
Diarrhea	86	(28.7)	86	(82.7)			
Abdominal pain	15	(5)	15	(14.4)			
Nausea	16	(5.3)	16	(15.4)			
Vomiting	18	(6)	18	(17.3)			
Loss of appetite	9	(3)	9	(8.65)			
Loss of taste	13	(4.3)	13	(12.5)			

*Significant

**Table 4:** Outcome of patients with COVID-19

Characteristics	All patients *N*=300	GI group *N*=104		Non-GI group *N*=196		*P* value	
Outcome *n*, (%)						0.18	
Discharge	267	(89.0)	96	(92.3)	171	(87.2)	
Died	33	(11.0)	8	(7.7)	25	(12.8)	
Length of stay mean ± SD	10.2 ± 6.6	10.2 ± 7.0		10.2 ± 6.4		0.97	
Duration from symptoms onset till outcome	17.7± 9.2	18.0± 8.0		17.5± 9.8		0.7	
Duration from symptoms onset till admission	7.5 ± 6.2	7.9± 3.8		7.4± 7.2		0.5	

Eighty-six patients presented with diarrhea that lasted from 1 to 21 days with a mean duration of 7±5.11 days. The daily frequency was 3.08±1.11 bowel movements. Not all patients with diarrhea had an accompanying fever. A total number of 57 patients (66.3%) had both diarrhea and fever occurred simultaneously, while 22 (25.6%) patients had diarrhea after fever, and only 1 (1.2%) before fever.

Vomiting was observed in 18 patients with an average of 2.89±1.32 vomitus daily. Abdominal pain was reported in 15 patients, where the majority (86.7%) described the pain as colicky in character.

### Laboratory and radiological findings

Patients with GI symptoms exhibited significantly lower total leucocytic count (TLC), neutrophil count, NLR, platelet count, and higher serum sodium than those with non-GI symptoms (*P* < 0.05). The mean ferritin level was higher in the GI group but had not reached a statistical significance (707.14±1063.52 vs 682.31±795.83 ng/ml, *P* = 0.82). Moreover, we found that the mean value of ALT was higher in patients with digestive symptoms but non-significant (43.16±36.23 vs 41.08±45.59 IU/L, *P* = 0.69). However, mean CRP, LDH, ESR, troponin, and D-dimer were lower but not statistically significant (Table 5).

**Table 5:** Laboratory and radiological findings in patients with COVID-19

	All patients *N*=300	GI group *N*=104	Non- GI group *N*=196	*P* value
TLC (10^9^/L)	7.62± 5.4	6.47± 3.24	8.24± 6.17	0.001*
Lymphocytes (10^9^/L)	1.36± 0.82	1.34± 0.84	1.37± 0.81	0.74
Neutrophils (10^9^/L)	5.29± 4.65	4.21± 2.68	5.87± 5.33	0.0001**
NLR	5.07± 5.04	4.33± 3.72	5.47± 5.59	0.036*
Hemoglobin (g/dl)	12.7± 1.96	12.91± 1.9	12.59± 1.99	0.17
Platelets (10^9^/L)	225.94± 91.74	209.15± 78.86	234.85± 96.91	0.01*
ALT (IU/L)	41.8± 42.52	43.16± 36.23	41.08± 45.59	0.69
AST (IU/L)	45± 40.29	41.69± 26.26	46.75± 46	0.3
T. Bilirubin (mg/dl)	0.7± 0.56	0.75± 0.75	0.67± 0.42	0.27
Albumin (g/dl)	3.47± 0.49	3.49± 0.47	3.45± 0.5	0.49
INR	1.1± 0.24	1.08± 0.19	1.11± 0.26	0.22
Creatinine (mg/dl)	1.07± 0.48	1.09± 0.55	1.06± 0.45	0.71
Na (mmol/L)	136.32± 4.5	137.16± 3.96	135.87± 4.71	0.02*
CRP (mg/dl)	50.12± 58.71	42.24± 51.57	54.32± 61.91	0.09
LDH (IU/L)	322.91± 188.82	312.42± 222.06	328.47± 168.91	0.49
Ferritin (ng/ml)	690.81± 894.77	707.14± 1063.52	682.31± 795.83	0.82
CK-Total (U/L)	154.62± 149.16	151.73± 170.32	156.15± 137.06	0.81
CK-MB (U/L)	25.3± 15.08	25.98± 14.99	24.93± 15.15	0.57
Troponin (pg/ml)	4.19± 49.64	2.15± 8.01	5.27± 61.17	0.61
ESR (mm/hr)	72.33± 36.84	66.88± 34.21	75.19± 37.92	0.06
D-dimer (mg/L)	1.09± 1.64	1.03± 1.17	1.13± 1.84	0.55
Radiological findings “HRCT”, *n* (%)				
Normal	6 (2.0)	4 (3.8)	2 (1.0)	0.58
CORAD 1	6 (2.0)	3 (2.9)	3 (1.5)	
CORAD 2	90 (30.0)	29 (27.9)	61 (31.1)	
CORAD 3	114 (38.0)	41 (39.4)	73 (37.2)	
CORAD 4	47 (15.7)	15 (14.4)	32 (16.3)	
CORAD 5	37 (12.3)	12 (11.5)	25 (12.8)	

*Significant

**Highly significant

Out of the total patients, 114 (38%) were CORADs category 3 in the HRCT chest at initial presentation, and 6 patients (2%) did not show any CT imaging features of COVID-19 pneumonia (Table 5).

### Outcome

Among patients with COVID-19, patients with gastrointestinal symptoms had lower mortality compared to those without (7.7% vs 12.8%, *P* = 0.18). Regarding the length of the hospital stay, there was no difference between the two groups (10.2±7 vs 10.2±6.4 days, *P* = 0.97). The duration from symptom onset to hospital admission was longer in the GI group (7.9±3.8 days) than in the patients with other symptoms (7.4±7.2 days) (Table 4).

## Discussion

Research on the disease has been accelerated because of its significant health and economic burden. Studies conducted at the early stage of the COVID-19 outbreak indicated that respiratory symptoms were common at the onset of the disease [20]. The fact that gastrointestinal manifestations can be a presentation of COVID-19 patients created a significant challenge in practice. In daily practice, a high index of suspicion for COVID-19 infection is currently considered while dealing with cases presenting with fever, anorexia, vomiting, and diarrhea instead of considering them as gastroenteritis [21]. Therefore, patients with digestive symptoms require special attention, and understanding the differences in clinical characteristics and outcomes between patients with and without these symptoms is critical.

Our study showed that the most common presentations of SARS-CoV-2 infection were fever (80%), cough (68.7%), and dyspnea (65%) which was consistent with previous studies [22–24]. Moreover, middle-aged, and elderly people were more susceptible to COVID-19, which may be contributed to the lower immunity in these populations.

Within our cohort, 104 (34.7%) patients presented with GI symptoms which is lower than the results reported by other studies conducted in the USA and China which were in the range of 50.5–61.3% [10, 12]. In contrast, this prevalence was higher than other studies conducted in China, Italy, and Egypt [7, 22, 24, 25].

Diarrhea was the most common GI symptom in our cohort (82.7%) which was similar to findings from Wang et al. [26]. Moreover, Shousha et al. [24], an Egyptian multicentric cohort study, agreed with our results where 86.67% had diarrhea. In contrast, a cross-sectional study conducted on 860 patients with COVID-19 infection reported that the most common GI symptom was vomiting (40.1%) followed by diarrhea (37.6%) [25].

There are several possible mechanisms for the gastrointestinal symptoms in COVID-19 patients. ACE-2, a receptor for SARS-CoV-2, is highly expressed not only in alveolar epithelial cells but also in the epithelium of the esophagus, small intestine, and colon suggesting that coronavirus has a tropism to the gastrointestinal tract [27]. Severe COVID-19 is known to cause a cytokine storm, in which over-activated lymphocytes secrete many cytokines such as interleukins (ILs); (IL2, IL6, IL7, etc.), and tumor necrosis factor (TNF). These proinflammatory cytokines can alter the gut-brain axis by getting their access via the vascular and lymphatic systems, resulting in systemic inflammatory response syndrome, which causes inflammation and damage of the digestive tract [28].

Moreover, the COVID-19 patients are commonly treated with antibiotics for secondary bacterial infections [29]. These microbial agents (such as fluoroquinolones and cephalosporins) can cause antibiotic-associated diarrhea as an adverse effect. Furthermore, antiviral agents such as hydroxychloroquine and remdesivir are increasingly used among these patients, and diarrhea is a common side effect of these drugs [28].

In our study, 99% of patients with digestive symptoms had moderate COVID-19. Shousha et al. [24] also reported that most patients with digestive symptoms (83.4%) had non-severe COVID-19. Our results disagree with previous reports [30, 31] that showed that GI symptoms were found to be associated with severe COVID-19. They speculated that GI symptoms are related to the degree of viral replication, with increased severity of the disease associated with high viral load.

Furthermore, this study noted that the presence of GI symptoms was associated with significantly lower TLC, neutrophil count, NLR, and platelet count, and no significant differences in serum ferritin, CRP, LDH, ESR, troponin, and D-dimer compared to patients without GI symptoms. In a multicenter study of 191 patients by Zhou et al. [32], the presence of GI symptoms was associated with significantly elevated CRP, ALT, and lower hemoglobin level when compared to patients without digestive symptoms. Moreover, Teima et al. [25] showed that the inflammatory markers (CRP, LDH, and ferritin) and D-dimer were significantly higher in patients with digestive symptoms. However, in the study by Redd and colleagues [12], there were no significant differences in the leucocytic count, hemoglobin, platelets, or liver function tests in groups with or without GI symptoms.

In consistent with the published literature [7, 24], no significant difference was detected in the mean level of transaminases among patients with and without digestive symptoms. In contrast, a meta-analysis by Wijarnpreecha et al. [33] and a cross-sectional study by Teima et al. [25] showed a higher level of transaminases in patients with GI symptoms.

Similar to previous reports [34–36], the presence of gastrointestinal symptoms although non-significant was associated with a longer duration from symptom onset till admission and lower mortality. These combined findings suggest that GI symptoms are associated with an indolent form of COVID-19. In contrast, Zheng et al. [22] demonstrated that the rate of clinical deterioration was significantly higher in the GI group than in the non-GI group (15.6% vs 10.1%, *P* = 0.032).

Limitations of our study are the lack of SARS-CoV-2 RNA in the stool of COVID-19 patients, so we did not determine the hypothesis that the severity of GI symptoms may be related to the presence of viral replication in stool since this test was not routinely performed in our institution.

## Conclusion

The prevalence of GI symptoms in our cohort of Egyptian patients was 34.7%. The significance of GI symptoms should not be underestimated in clinical practice. We recommend physicians to be aware of COVID-19 infection in patients with acute GI illness. We also recommend that appropriate personal protective equipment (PPE) should be worn by all gastroenterologists.

## Data Availability

The datasets used and analyzed during the current study are available from the corresponding author upon reasonable request.

## Abbreviations

COVID-19: Coronavirus disease 2019
GI: Gastrointestinal
SARS-CoV-2: Severe acute respiratory coronavirus 2
WHO: World Health Organization
ACE-2: Angiotensin-converting enzyme 2
PCR: Polymerase chain reaction
ICU: Intensive care unit
CBC: Complete blood count
NLR: Neutrophil lymphocyte ratio
ALT: Alanine aminotransferase
AST: Aspartate aminotransferase
INR: International normalization ratio
Na: Sodium
K: Potassium
CRP: C-reactive protein
LDH: Lactate dehydrogenase
ESR: Erythrocyte sedimentation rate
HRCT: High-resolution computed tomography
CORADs: COVID-19 Reporting and Data System
TLC: Total leucocytic count
ILs: Interleukins
TNF: Tumor necrosis factor
PPE: Personal protective equipment
